# New evidence that vitamin D prevents headache: a bidirectional two-sample Mendelian randomization analysis

**DOI:** 10.3389/fneur.2024.1423569

**Published:** 2024-07-26

**Authors:** Haibing Xiong, Ran Jiang, Lingzhi Xing, Jiaojiao Zheng, Xinhong Tian, Jiajie Leng, Xin Guo, Shi Zeng, Haofeng Xiong, Jianhong Huo, Letai Li

**Affiliations:** ^1^Banan Hospital Affiliated to Chongqing Medical University, Chongqing, China; ^2^The First College of Clinical Medicine, Chongqing Medical University, Chongqing, China; ^3^Faculty of Pediatrics, Chongqing Medical University, Chongqing, China; ^4^The Second College of Clinical Medicine, Chongqing Medical University, Chongqing, China; ^5^The First Affiliated Hospital of Chongqing Medical University, Chongqing, China

**Keywords:** vitamin D, headache, Mendelian randomisation study, single nucleic acid polymorphism, prevention

## Abstract

**Background:**

Previous observational clinical studies and meta-analyses have yielded inconsistent results regarding the relationship between vitamin D and headache, and the causal relationship remains unclear. The aim of this study was to investigate the causal relationship between vitamin D and headache by bidirectional two-sample Mendelian randomisation (MR) analysis.

**Methods:**

The relationship between high levels of vitamin D and headache was investigated by two-sample MR analysis using publicly available genome-wide association study (GWAS) data. The primary method was inverse variance weighting (IVW), and secondary methods were weighted median and MR-Egger methods. No heterogeneity or horizontal multidirectionality was found in the MR results. The robustness and validity of the findings were assessed using the leave-behind method.

**Results:**

A significant causal relationship was found between high vitamin D levels and headache using the IVW method (OR = 0.848; *p* = 0.007; 95% CI = 0.752–0.956). However, in a reverse analysis, no evidence of a causal relationship between headache and high levels of vitamin D was found using the IVW method (OR = 1.001; *p* = 0.906; 95% CI = 0.994–1.006). Our MR analyses showed no significant horizontal multidimensionality or heterogeneity (*p* > 0.05). Sensitivity analyses confirmed that MR estimates were not affected by single nucleotide polymorphisms (SNPs). Confirmation that our results are robust and valid has been obtained by the leave-one-out method.

**Conclusion:**

Our study suggests that high levels of vitamin D prevent the risk of headache. However, there is no evidence of a causal relationship between headache and high levels of vitamin D. Vitamin D may reduce the risk of headache.

## Introduction

Vitamin D is a fat-soluble vitamin that plays several important roles in the body (1). It is mainly synthesised through the skin in the presence of sunlight and can also be ingested through food. Vitamin D promotes the absorption and utilisation of calcium and contributes to normal bone development and maintenance. It plays an important role in immune regulation, cell differentiation and inflammatory responses (2–5). In recent years, studies have increasingly focused on the role of vitamin D in neuropathic pain. Vitamin D receptors are widely distributed in the central and peripheral nervous system, suggesting that vitamin D may play a role in normal nervous system function and nociceptive modulation (6). Several studies have shown an association between vitamin D deficiency and the onset and exacerbation of headache, and that vitamin D supplementation can help alleviate the symptoms of headache (7, 8).

Headache is a common neurological symptom that manifests as pain or discomfort in different areas of the head (9). Its etiology is complex and diverse, including intracranial lesions, vascular abnormalities, infection, inflammation, and metabolic disorders (10). Headache can be divided into two categories: primary and secondary; the former, such as migraine and tension headache, are mostly associated with genetic, endocrine, and environmental factors; the latter is caused by specific diseases, such as brain tumour and cerebrovascular disease. The severity, frequency of attacks and accompanying symptoms of headache vary from person to person and affect the daily life and work of patients (11).

Preliminary studies have investigated the correlation between vitamin D and headache. Some studies have shown that people with lower levels of vitamin D are at greater risk of headaches and have more severe pain (12). In addition, vitamin D supplementation appears to reduce headaches and improve patients’ quality of life (13, 14). Although some relevant studies have shown a correlation between vitamin D and headache, it is difficult to establish a specific causal relationship between the two.

Mendelian randomization (MR) analysis is an emerging statistical method that uses genetic variation as an instrumental variable to explore potential causal relationships between exposure factors and disease. Mendelian randomisation analysis provides a classification effect similar to that of a randomised controlled trial (RCT) by randomly classifying genetic alleles during sperm-egg binding (15). However, MRI analyses provide stronger causal inferences than traditional observational studies and are effective in controlling for the effects of confounding factors (16). Furthermore, there is a lack of magnetic resonance studies exploring the potential causal relationship between vitamin D and headache, suggesting that further research is needed in this area.

The aim of this study was to investigate the potential causal relationship between vitamin D and headache. By integrating existing genome-wide association study (GWAS) data, we aimed to assess the effect of vitamin D levels on the risk of headache onset. This will provide a scientific basis for the development of targeted prevention and treatment strategies.

## Methods

### The study design

**Figure 1:**
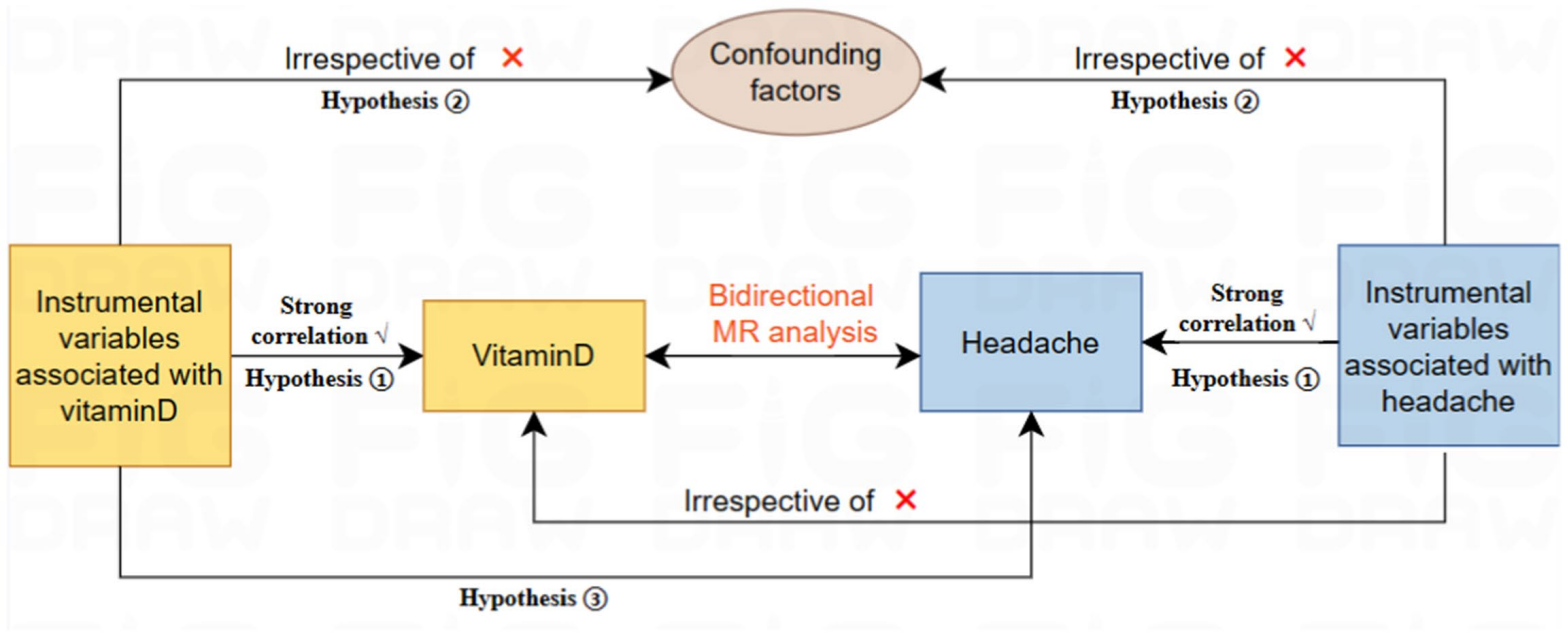
Flowchart of Mendelian randomization.

For MR analysis requires a valid instrumental variable (IV) that satisfies three key assumptions to obtain reliable results. Firstly, the IV must be strongly associated with the exposure. Secondly, the IV must be independent of any confounding factors that may affect the exposure and outcome. Finally, the IV must affect the outcome solely through the exposure (17). The study consisted of several core steps, including the use of multiple MR methods (IVW, WM, MR Egger), multiplicity assessment, and heterogeneity and sensitivity analyses. These steps were taken to select genetic IVs associated with exposure and to examine the association between vitamin D levels and headache. To further investigate the causal relationship between vitamin D and headache, a bidirectional two-sample MR study was conducted (see Figure 1). To reduce bias resulting from population stratification and racial differences, we only selected samples from the same racial group. Furthermore, this study followed the latest guidelines for MR in epidemiological studies (STROBE-MR) (18).

### Data sources

The genetic association data for this study were obtained from the IEU Open GWAS database.^1^ The low level vitamin D dataset (ieu-b-4812) comprises 441,291 participants with a total of 16,668,957 SNPs. The dataset on headaches (finn-b-R18_HEADACHE1) was obtained from the Finngen Consortium and includes 13,345 cases and 172,999 controls.

### The selection of IV

To fulfill the initial step of the first hypothesis, we identified single nucleotide polymorphisms (SNPs) that were significantly associated with exposure based on stringent criteria (*p* < 5 × 10^–8^) and independence (*r*^2^ < 0.001, kb = 10,000) in order to select genetic IVs that are strongly associated with exposure. However, in a reverse MR analysis with headache as the exposure, no SNPs significantly associated with exposure at *p* < 5 × 10^–8^ were found. The significance level was set at *p* < 5 × 10^–6^ for the reverse MR analysis. Furthermore, all SNPs with palindromes and ambiguities were excluded to ensure consistent effect alleles between the exposure and outcome datasets. To evaluate the strength of the instrumental variable (IV), we calculated the *F* statistic value using the formula *F* = (*N* − 2) * *R*^2^/(1 − *R*^2^) (19). An *F* value greater than 10 indicates a low risk of weak IV bias and avoids weak instrumental bias (20).

### Statistical analysis

This study used three methods, namely inverse variance weighted (IVW) and MR-Egger, weighted median (WM), to establish the causal relationship between vitamin D and headache. IVW is the dominant method and produces the highest statistical efficacy when all instrumental variables (IVs) are validated tools (20). Criteria for establishing causality include significant results in IVW analyses. The results of WM and MR-Egger analyses should align with those of IVW analyses (21–23).

For sensitivity analyses, we used the MR-Egger intercept to determine the presence of pleiotropy. Intercept values close to 0 and *p*-values greater than 0.05 indicate no horizontal pleiotropy (24). We then used Cochran’s *Q*-test to quantify the heterogeneity of the IVW estimates. A p-value greater than 0.05 indicates no heterogeneity (25). The results of the heterogeneity analysis are shown in Table 1. Additionally, we ran the MR-PRESSO test to check for outliers (see Supplementary material). If any outliers were identified, they were removed, and the MR effect was re-evaluated. To ensure the robustness of the results, we also conducted leave-one-out analyses to examine the impact of individual SNPs on the overall causal effect (26). Final funnel plots were used to assess the symmetry of the selected SNPs, while forest plots were used to evaluate the reliability and heterogeneity of chance estimates. Scatter plots were used to visualise the relationship between exposure and outcome. Please refer to the Supplementary material for additional details.

**Table 1:** The heterogeneity and sensitivity of omega-3/omega-6 fatty acids and cerebrovascular disease after removal unqualified IVs.

Exposure	Outcome	*n*SNP	MR Egger intercept		Cochran’s heterogeneity			
			Intercept value	*p*	IVW-*Q* value	*p* (IVW)	Egger-*Q* value	*p* (Egger)
VD	Headache	99	0.002664378	0.3248348	121.0626	0.05703579	119.8526	0.05772855
Headache	VD	116	0.000534979	0.3054296	127.4209	0.2019028	126.2471	0.2039701

MR, Mendelian randomization; IVW, inverse variance weighted; *n*SNP, number of single nucleotide polymorphisms.

The methodology used in the inverse MR analysis was the same as described above, which involved using SNPs associated with headache to investigate the causal effect of headache and vitamin D. The results of the inverse MR analysis were presented in RStudio. The entire analysis was conducted in R Studio (version 4.3.0) using the “TwoSampleMR” and “MRPRESSO” software packages.

## Results

**Figure 2:**
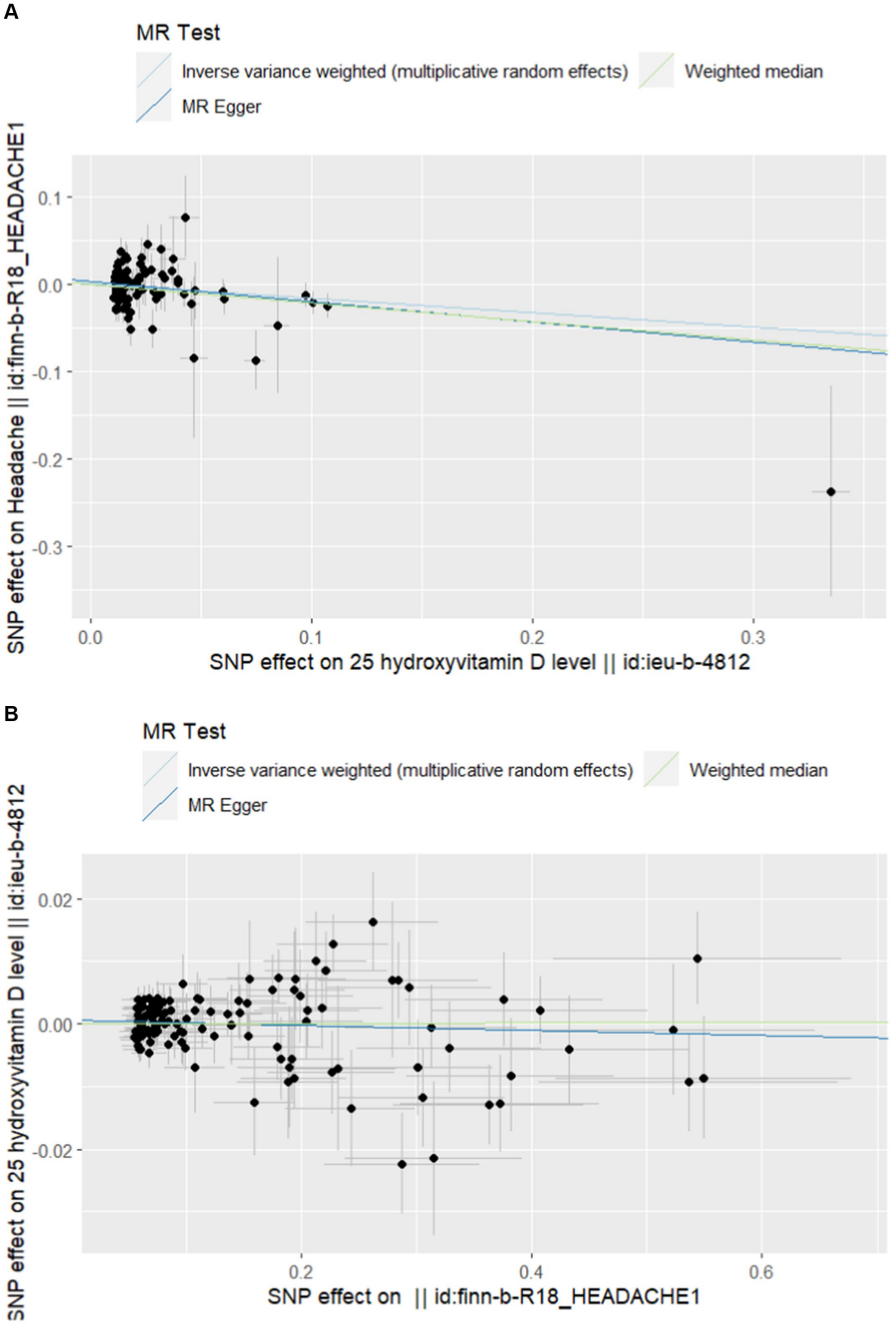
**(A)** Scatterplot of forward Mendelian randomization analysis. **(B)** Scatter plot of reverse Mendelian randomization analysis.

Finally, we included 101 and 121 SNPs in the exposure and outcome datasets, respectively. Two and five SNP deleted due to palindromes, respectively. As shown in Table 2, the results of IVW analysis indicated that high-level vitamin D were significantly associated with an decreased risk of headache (OR = 0.848; *p* = 0.007; 95% CI = 0.752–0.956). This finding was also supported by WM and MR Egger. The forest plots of SNP effect sizes for each phenotype in the forward analyses are presented in Figure 2. No horizontal pleiotropy was observed for any of the phenotypes (MR Egger intercept, *p* > 0.05). After removing the palindromic SNPs, there was no heterogeneity observed among the exposure-associated SNPs. The MR-Egger intercept test results indicated no outliers or evidence of horizontal pleiotropy. Additionally, leave-one-out analyses revealed that no single SNP had a potential effect on MR estimates. The funnel plots were essentially symmetrical in both the forward and reverse MR analyses, suggesting no directional horizontal pleiotropy in the selected variables. The Supplementary material display scatter plots, funnel plots, and leave-one-out methods for forward analyses, which can demonstrate the absence of outliers that may affect causality.

**Table 2:** Results of forward and reverse Mendelian randomization analysis.

Exposure	Outcome	SNPs	OR	*p*-value	Low	High
*VD*						
MR Egger	Headache	99	0.793	0.012	0.664	0.947
Weighted median	Headache	99	0.809	0.009	0.689	0.949
IVW	Headache	99	0.848	0.007	0.752	0.956
*Headache*						
IVW	VD	116	0.996	0.437	0.986	1.006
MR Egger	VD	116	1.001	0.953	0.991	1.008
Weighted median	VD	116	1.001	0.906	0.994	1.006

All statistical tests were two-sided. *p* < 0.05 was considered significant. *n*SNP, number of single nucleotide polymorphisms; OR, odds ratio; IVW, inverse variance weighted; SAH, subarachnoid hemorrhage; ICH, Intracerebral hemorrhage; IS, ischemic stroke.

In the reverse MR analysis, none of the causal relationships between headache and vitamin D were significant when headache was considered as exposure (OR = 1.001; *p* = 0.906; 95% CI = 0.994–1.006). Table 2 and Figure 2 provided further details. The statistical analysis indicated that there was no significant causal relationship between headache and vitamin D. The Supplementary material include scatterplots, funnel plots, and leave-one-out methods for inverse analyses.

## Discussion

This is the first study to explore a bidirectional causal relationship between vitamin D and headache using MR analysis. The results of the MR analysis showed that high levels of vitamin D significantly decreased the risk of headache. Furthermore, reverse MR studies did not find any evidence of a causal relationship between genetically predicted headache and vitamin D levels.

The relationship between vitamin D and headaches remains inconclusive, despite numerous studies exploring the topic. A case-control study conducted in Egypt found a significant vitamin D deficiency in migraine patients, which can significantly impact the character, duration, frequency, and severity of headache attacks (27). Similarly, a study in Turkish children also found a possible link between vitamin D deficiency and headaches (28). A cross-sectional descriptive study conducted in Norway confirmed that patients with headaches had lower average vitamin D levels compared to patients with other types of pain symptoms. Specifically, patients with headaches had a higher incidence of vitamin D deficiency (29). Quintero-Fabián et al. (30) found that vitamin D acts as an immunomodulatory hormone to reduce neuroinflammation and prevent headaches as a neurological disorder. Recent literature suggests that migraine sufferers may have a vitamin D deficiency, and taking vitamin D alongside conventional medication may reduce the frequency of migraine attacks (31). However, further verification of these results by other methods is necessary.

It is important to note that not all research findings support a link between vitamin D and headache. A randomized controlled trial conducted in Norway found that the use of vitamin D supplements did not have a significant effect on the occurrence and extent of pain or headache (32). Furthermore, a recent meta-analysis did not find any relationship between cluster headache and the three single nucleotide polymorphisms of the vitamin D receptor gene (33). The researchers also noted the lack of articles exploring the relationship between vitamin D and headaches. The relationship between vitamin D and headaches remains a controversial issue for objective reasons.

The varying results could be attributed to the fact that the majority of the studies were observational or meta-analyses and lacked the support of prospective randomized studies. Observational studies have inherent limitations, such as methodological flaws, selection bias, and insufficient adjustment for confounders, which make it difficult to establish a clear causal link. Mendelian randomization is a research methodology that can reveal the causal relationship between exposure and outcome by using genetic variation as an instrumental variable. This approach avoids the influence of non-heritable environmental factors. The study found a significant causal relationship between vitamin D and headache using two-sample MR analysis. A reverse MR study further confirmed this finding’s robustness. Our study did not find significant levels of pleiotropy or heterogeneity, which increases the credibility and reliability of our findings.

However, it is worth noting that MR studies have limitations. Firstly, existing databases lack data on different levels of vitamin D, making it difficult to explore the specific association between vitamin D levels and headache. Secondly, as our GWAS data is primarily derived from European populations, our findings may exhibit some racial or geographic bias and require further validation in other ethnic groups. It is expected that more high-quality studies and data will be published in the future to provide additional insight into the relationship between vitamin D and headache. Further research and technological advances may reveal the exact link between the two, leading to new ideas and approaches for preventing, testing, and treating headache.

## Data availability statement

The datasets presented in this study can be found in online repositories. The names of the repository/repositories and accession number(s) can be found in the article/Supplementary material.

## Ethics statement

Ethical review and approval was not required for the study on human participants in accordance with the local legislation and institutional requirements. Written informed consent from the patients/participants or patients/participants’ legal guardian/next of kin was not required to participate in this study in accordance with the national legislation and the institutional requirements.

## Author contributions

HiX: Funding acquisition, Investigation, Writing – original draft, Writing – review & editing, Methodology. RJ: Software, Writing – review & editing. LX: Investigation, Software, Writing – original draft, Writing – review & editing. JZ: Software, Writing – original draft, Writing – review & editing. XT: Investigation, Writing – original draft. JL: Methodology, Writing – original draft. XG: Data curation, Writing – original draft. SZ: Methodology, Writing – original draft. HoX: Formal analysis, Writing – original draft. JH: Writing – original draft. LL: Data curation, Formal analysis, Methodology, Supervision, Writing – original draft, Writing – review & editing, Investigation.

## Footnotes

^1^ https://gwas.mrcieu.ac.uk/

## Supplementary material

SUPPLEMENTARY FIGURE S1 **(A)** Forward Mendelian randomized leave-one graph. **(B)** Reverse Mendelian randomized leave-one graph.

SUPPLEMENTARY FIGURE S2 **(A)** Funnel plot for forward Mendelian randomization. **(B)** Funnel plot for reverse Mendelian randomization.

SUPPLEMENTARY FIGURE S3 **(A)** Forward Mendelian randomization of forest graphs. **(B)** Reverse Mendelian randomization of forest graphs.
