# Supplementary figures and images for: New evidence that vitamin D prevents headache: a bidirectional two-sample Mendelian randomization analysis
Source: Front Neurol. 2024 Jul 26;15:1423569. doi: 10.3389/fneur.2024.1423569 (PMC11310154; doi:10.3389/fneur.2024.1423569)

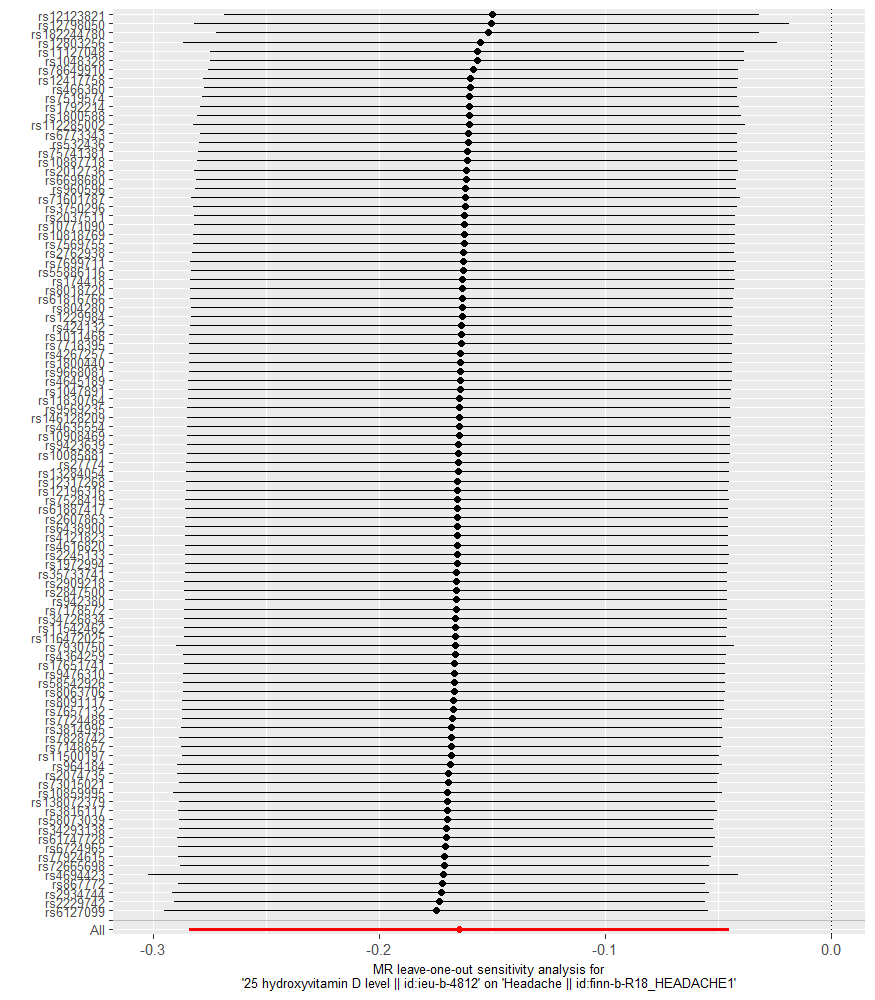

Supplement: SUPPLEMENTARY FIGURE S1 — (A) Forward Mendelian randomized leave-one graph. (B) Reverse Mendelian randomized leave-one graph. [file Image_1.png]

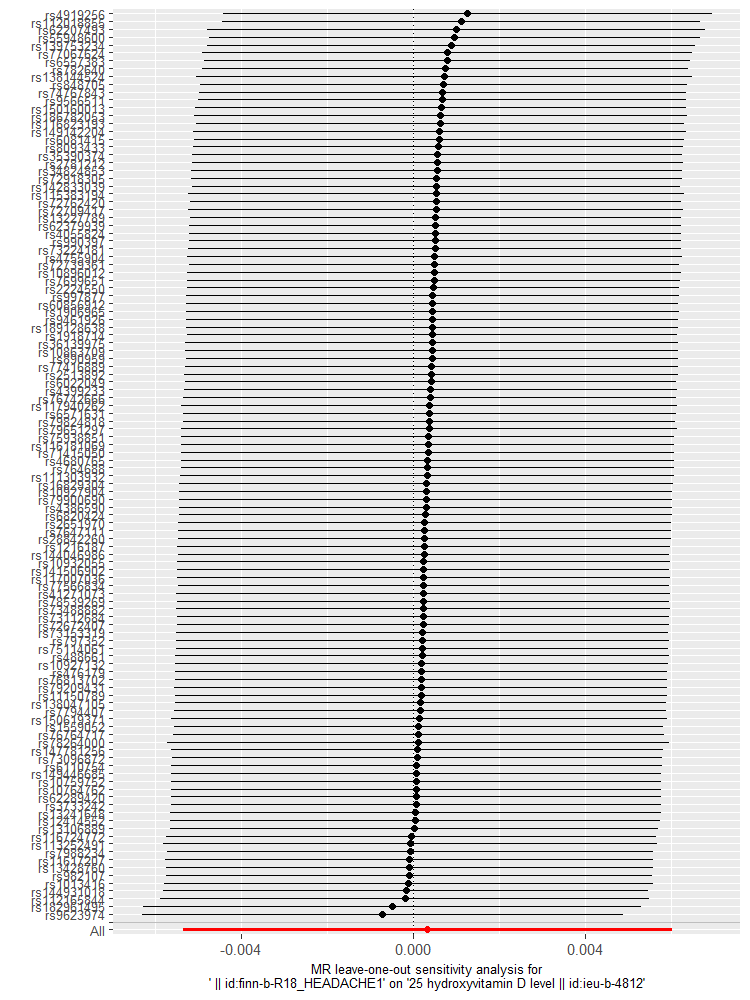

Supplement: Supplementary file 2 [file Image_2.png]

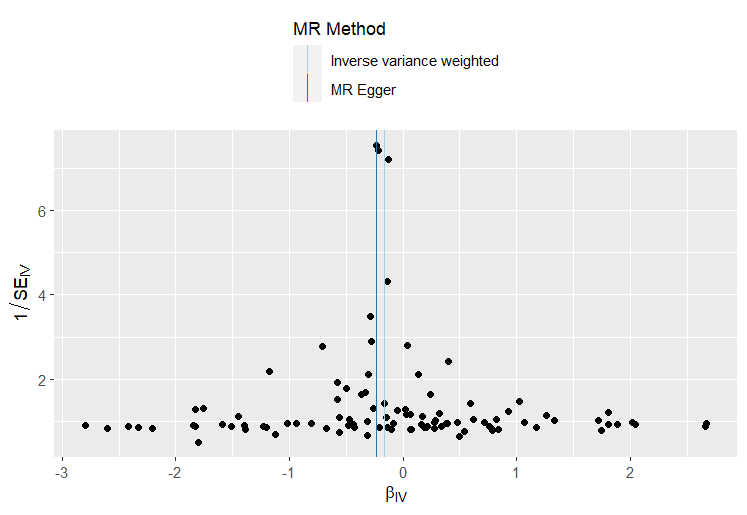

Supplement: SUPPLEMENTARY FIGURE S2 — (A) Funnel plot for forward Mendelian randomization. (B) Funnel plot for reverse Mendelian randomization. [file Image_3.png]

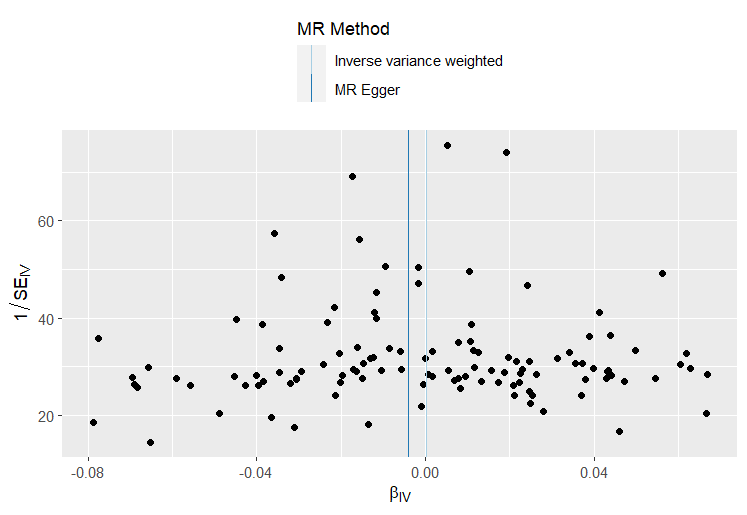

Supplement: Supplementary file 4 [file Image_4.png]

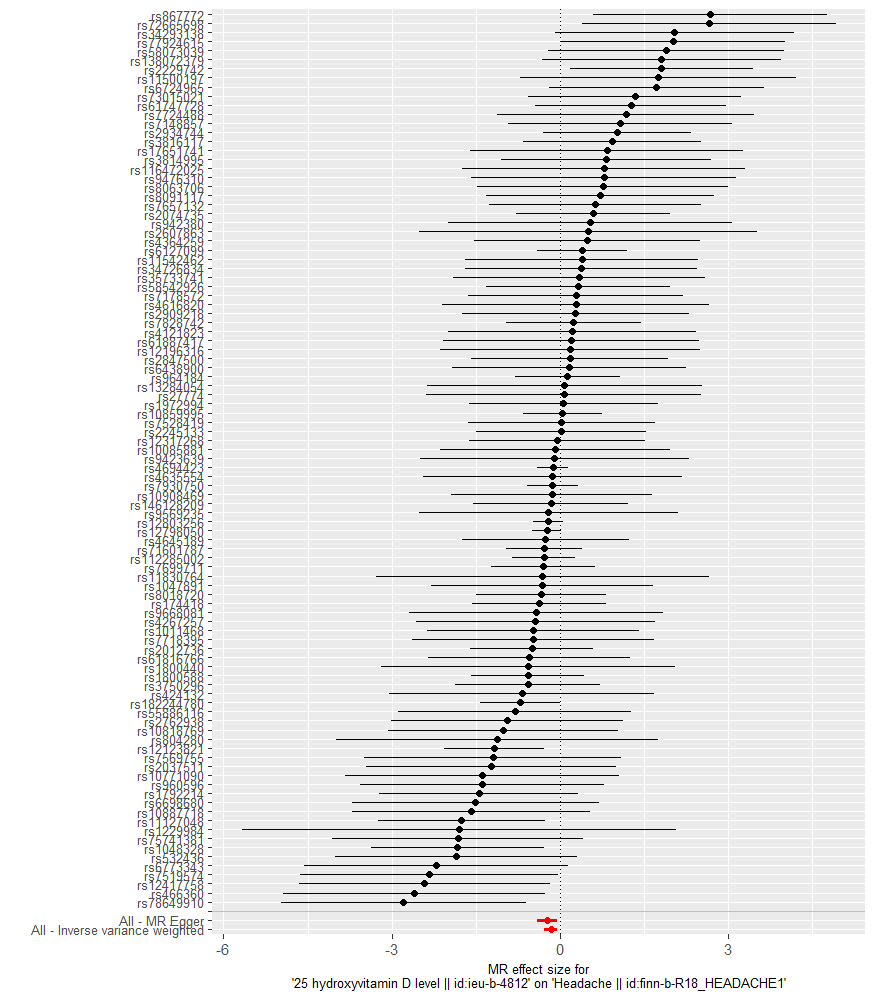

Supplement: SUPPLEMENTARY FIGURE S3 — (A) Forward Mendelian randomization of forest graphs. (B) Reverse Mendelian randomization of forest graphs. [file Image_5.png]

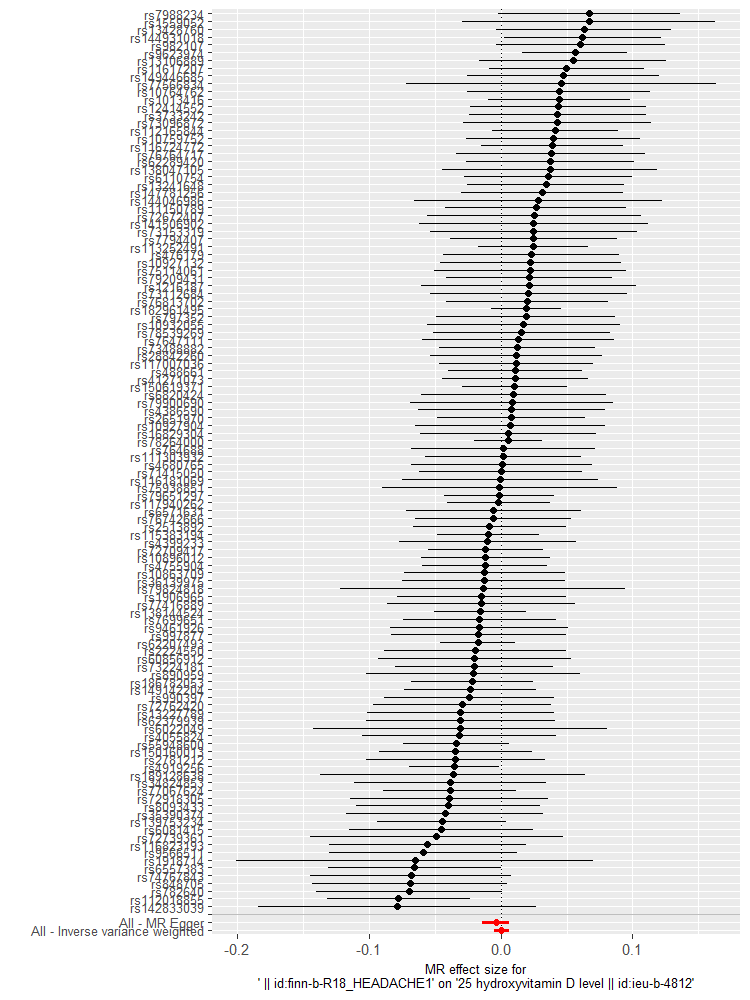

Supplement: Supplementary file 6 [file Image_6.png]
